# Six in ten children with epilepsy visiting the University of Gondar comprehensive specialized hospital were undernourished: a cross-sectional study

**DOI:** 10.1186/s40795-022-00606-8

**Published:** 2022-10-12

**Authors:** Geta Bayu Genet, Nahom Worku Teshager, Alemayehu Teklu Toni

**Affiliations:** Department of Pediatrics and Child Health, School of Medicine, College of Medicine and Health Sciences, University of Gondar, Gondar, Ethiopia

**Keywords:** Undernutrition, Malnutrition, Epilepsy, Children, Gondar, Ethiopia

## Abstract

**Background:**

The burden of undernutrition among children with epilepsy in low- and middle-income countries is not well studied. This study aimed to assess the magnitude of undernutrition and associated factors among children with epilepsy at the University of Gondar Comprehensive and Specialized Hospital, Northwest Ethiopia.

**Method:**

A single-center cross-sectional study was conducted on 239 epileptic children with epilepsy visiting the University of Gondar Comprehensive Specialized Hospital pediatric neurology clinic from June 2021 to September 2021. A pre-tested, researcher-administered questionnaire and medical record review were used for data collection. We included all participants who fulfilled the inclusion criteria. We did anthropometric measurements and defined undernutrition based on the world health organization criteria. Binary and multivariable logistic regressions were employed to determine factors associated with undernutrition. The statistical association between dependent and independent variables was declared at p-value of ≤ 0.05.

**Result:**

The mean(+/-SD) age was 9.38 ± 0.29 years, with a male to female ratio of 1.8: 1, and school-age children account for 35.6%. The overall magnitude of undernutrition was 141(59%) of which 89(63.1%) had moderate to severe stunting, 91(64.5%) moderate to severe wasting, and 39(27.7%) had both. Being male (AOR = 1.96, 95%CI, 1.05–3.69), low paternal level of education (AOR = 1.88, 95%CI, 1.01–3.50), presence of delay in motor development (AOR = 5.91,95%CI, 1.55–22.49), and gum hyperplasia (AOR = 0.32,95%CI, 0.12–0.81), were significantly associated with undernutrition.

**Conclusion:**

The magnitude of undernutrition among children with epilepsy was high. Male sex, low paternal level of education, presence of delay in motor development, and gum hyperplasia were significantly associated with undernutrition. Therefore, nutritional screening and intervention are recommended to be part of routine epileptic care.

## Background

Undernutrition is one of the major underlying causes of death in children and often complicates chronic illnesses and affects the treatment outcome. It is also associated with longer negative health consequences such as developmental delay, recurrent infections, neurocognitive problems, and generational defects[1–3]. According to the 2016 global report, about 45% of under-five mortality was associated with undernutrition[4].

Epilepsy is the top cause of neurology clinic visits worldwide, and its cognitive, psychological, and social consequences affect all ages, races, social classes, and geographic locations[5]. Even though primary undernutrition is an important public health problem, one should not lose sight of the fact that undernutrition often happens secondary to an underlying chronic illness. Undernutrition jeopardizes these deleterious consequences of the epileptic disorder leading to poor quality of life and increased mortality and morbidity[6, 7]. Undernutrition among children with epilepsy could be due to inadequate intake as a result of vomiting, chewing and swallowing difficulty, and cognitive impairment.

In patients with poorly controlled epilepsy, disturbing quality of life for patients and guardians is worth screening for and managing undernutrition, especially in developing countries. Undernutrition negatively affects seizure control possibly by lowering the seizure threshold due to biochemical alterations induced by undernutrition such as electrolyte abnormalities and hypoglycemia as well as the immunological vulnerability of undernourished children which predisposes them to various types of infections. Several studies showed that undernutrition is highly prevalent in children with epilepsy. The magnitude and factors associated with undernutrition in children with epilepsy are believed to vary by socioeconomic and demographic factors status including age and sex, comorbid condition, clinical presentation, and treatment-related factors [8–10].

The risk of undernutrition depends on the severity and type of underlying neurologic disorder that affects ambulation, cognitive status, seizure type, and the number and type of anti-epileptic drugs used. Moreover, epilepsy in developing countries is considered as an evil spirit, and children with epilepsy are usually socially isolated and neglected. Some food items are excluded from the diet of children with epilepsy because they are thought to provoke seizure attacks. Thus, the knowledge, attitude, and practice of guardians towards feeding children with epilepsy can also affect the nutritional status in such population[8–10].

Evidence on the magnitude and associated factors of undernutrition in children with epilepsy is scarce in developing countries. There are limited studies in Ethiopia. This study aimed to assess the magnitude and associated factors of undernutrition among children with epilepsy visiting the university of Gondar comprehensive specialized hospital.

## Method

**A single-center** cross-sectional study was conducted at the pediatric neurology clinic of the University of Gondar Comprehensive Specialized Hospital (UOGCSH). This is the only tertiary care hospital in Northwest Ethiopia. The pediatric neurology clinic delivers care for a total of 372 children with epilepsy which accounts for 40% of the chronic care burden on the pediatric side. The team composition is limited to Medical Interns, General practitioners, pediatric residents, nurses, and general pediatricians.

All children with epilepsy who visited the pediatric neurology clinic at UOGCSH during the data collection period were considered as the study population. Children who had major surgery other than Epilepsy surgery or surgery for traumas sustained during seizure attacks within the past three months were excluded. The sample size for this study was determined using a single population proportion of p = 50% with a 5% margin of error with correction for < 10,000 population; the sample size became 217, and after adding 10% contingency, it became 239 and we took them with consecutive sampling technique.

Data were collected by trained physicians using a structured questionnaire. Informed consent was taken from parents, legal guardians, or assent was taken from the patient when applicable. Ethical clearance was obtained from the school of medicine Ethical Review Committee.

Socio-demographic variables including the age of the child, parental age, sex, family size, residence, parental level of education, parental occupation, and maternal marital status were included in the questionnaire. Nutritional and dietary history, presence of comorbid illness, and clinical data of underlying disorder; age at diagnosis, duration of illness, adherence, drug side effect, frequency of seizure, and frequency of admission were also assessed.

**Children** implies to any human being who is less than 18 years old[11]. **Epilepsy** was defined as the presence of two or more seizures 24 h apart or a single seizure with possibility of recurrence. **Well-controlled seizure**; Maximum of one seizure episode in the last 3 months after treatment initiation. **Good control seizure**; Maximum of three seizure episodes in the last 3 months after treatment initiation[12]. **Poorly controlled epilepsy**; Maximum of nine and a minimum of four seizure episodes in the last three months after treatment initiation. **Uncontrolled epilepsy**; Ten and above seizure episodes in the last three months after the start of treatment. **Excellent Adherent**; if the patient is taking > 90% of monthly prescribed medication [12] **Good adherence**; if the patient is taking > 85% of monthly prescribed medication **Poor adherence**; if the patient is taking < 85% of monthly prescribed medication.

Nutritional status was assessed using body mass index (BMI) and mid-upper arm circumference (MUAC), height-for-age (HFA), and measurements were interpreted in accordance with World Health Organization (WHO) standards. For the anthropometric measurements, data analysis was performed using Emergency Nutrition Assessment (ENA) for SMART 2007 software.

Data were entered into EpiData V.4.6 and exported to STATA version 15.1 for cleaning and analysis after it was double-checked for consistency and completeness. Descriptive statistics like mean, median, and proportions were computed to summarize baseline socio-demographic and clinical characteristics. A p-value of less than 0.2 was used to select candidate variables for multivariable analysis. A binary logistic regression model was fitted to identify factors associated with undernutrition. Adjusted odds ratio with 95% confidence interval (CI) was calculated and variables with a p-value less than 0.05 in the multi-variable analysis were considered to declare factors associated with undernutrition.

Model fitness was tested using the Hosmer Lemeshow goodness of fit test (p = 0.2208).

## Result

### Socio-demographic characteristics

We studied two hundred thirty-nine children with epilepsy who visited the pediatric neurology clinic during the study period with a response rate of 100%. The mean (+/- SD) age was 9.38 ± 0.29 years, with a male to female ratio of 1.8: 1, and school-age children account for 35.6%. About half (50.6%) of the mothers of the participants were housewives, and two-thirds (77.4%) were married. Nearly half (45.6%) of the fathers of participants were unable to read/write. (Table 1)

**Table 1:** Sociodemographic characteristics of children with epilepsy at University of Gondar comprehensive specialized hospital (n = 239)

Variables	Category	Frequency	Percent	
Age	≤ 5years 5-10 years 10-15years 15-18 years	55 85 68 37	23.01 35.56 28.45 12.97	
Sex	Male Female	151 88	63.18 36.82	
Address	Urban Rural	142 97	59.41 40.59	
Maternal age	<=30 years 30-45 years 45-60years > 60years	74 148 15 2	30.96 61.92 6.28 0.84	
Maternal education	No formal education	118	49.37	
	Primary and above	121	50.63	
Maternal occupation	Employed Housewife Merchant Farmer daily laborer Other	38 121 22 35 19 4	15.90 50.63 9.21 14.64 7.95 1.67	
Paternal age	<=30 years 30–45 years 45–60 years > 60 years	22 137 63 17	9.21 57.32 26.36 7.11	
Paternal occupation	Employed Merchant Farmer daily laborer Other	64 35 103 28 9	26.78 14.64 43.10 11.72 3.77	
Paternal education	No formal education	105	43.93	
	Primary and above	134	56.07	

### Clinical and treatment-related characteristics

The mean age at diagnosis of epilepsy was 5.28 ± 0.27 years. The majority of children (65%) were on phenytoin. About 82.9%, 6.3%, 1.3%, and 9.6% of participants were having excellent, good, fair, and poor seizure control respectively. Forty-four (18.4%) of participants were having comorbid illnesses, of which 66% had cerebral palsy (Tables 2 and 3).

**Table 2:** Clinical characteristics of children with epilepsy at the University of Gondar comprehensive specialized hospital (N = 239)

Variable	Category	frequency	Percent
Developmental delay	Yes	77	32.22
	No	162	67.78
Age at diagnosis	<=1year	48	20.08
	1-5years	93	38.91
	5–10 years	58	24.27
	> 10 years	40	16.74
Duration before diagnosis of epilepsy	< 1 year	156	65.27
	1-5years	74	30.96
	> 5years	9	3.77
Duration after diagnosis of epilepsy	< 1 year	65	27.2
	1–5 years	124	51.88
	5–10 years	37	15.48
	> 10 years	13	5.44
Seizure control	Well-control	183	76.57
	Good control	15	6.28
	Poor control	18	7.53
	Uncontrolled	23	9.26
Frequency of daily food intake	<=2 times	16	6.69
	=3 times	184	76.99
	>=4 times	39	16.32
Other comorbidities	No	195	81.59
	Yes	44	18.41
Admission within the past 6 month	No	214	89.54
	Yes	25	10.46

**Table 3:** Treatment and follow-up of children with epilepsy at the University of Gondar comprehensive specialized hospital (N = 239)

Variable	Category	frequency	Percent
The average frequency of follow-up	Every 1 month	147	61.51
	Every 2 month	77	32.22
	Every 3 month	13	5.44
	Greater than 3 months	2	0.84
Adherence (medication and follow-up)	Excellent	223	93.31
	Good	12	5,02
	Poor	4	1.67
Phenytoin	No	82	34.31
	Yes	157	65.69
Phenobarbital	No	140	58.58
	Yes	99	41.42
Carbamazepine	No	234	97.91
	Yes	5	2.09
Valproic acid	No	193	80.75
	Yes	46	19.25
Presence of adverse effect	No	186	77.82
	Yes	53	22.18

Magnitude of undernutrition among epileptic children.

**Fig. 1:**
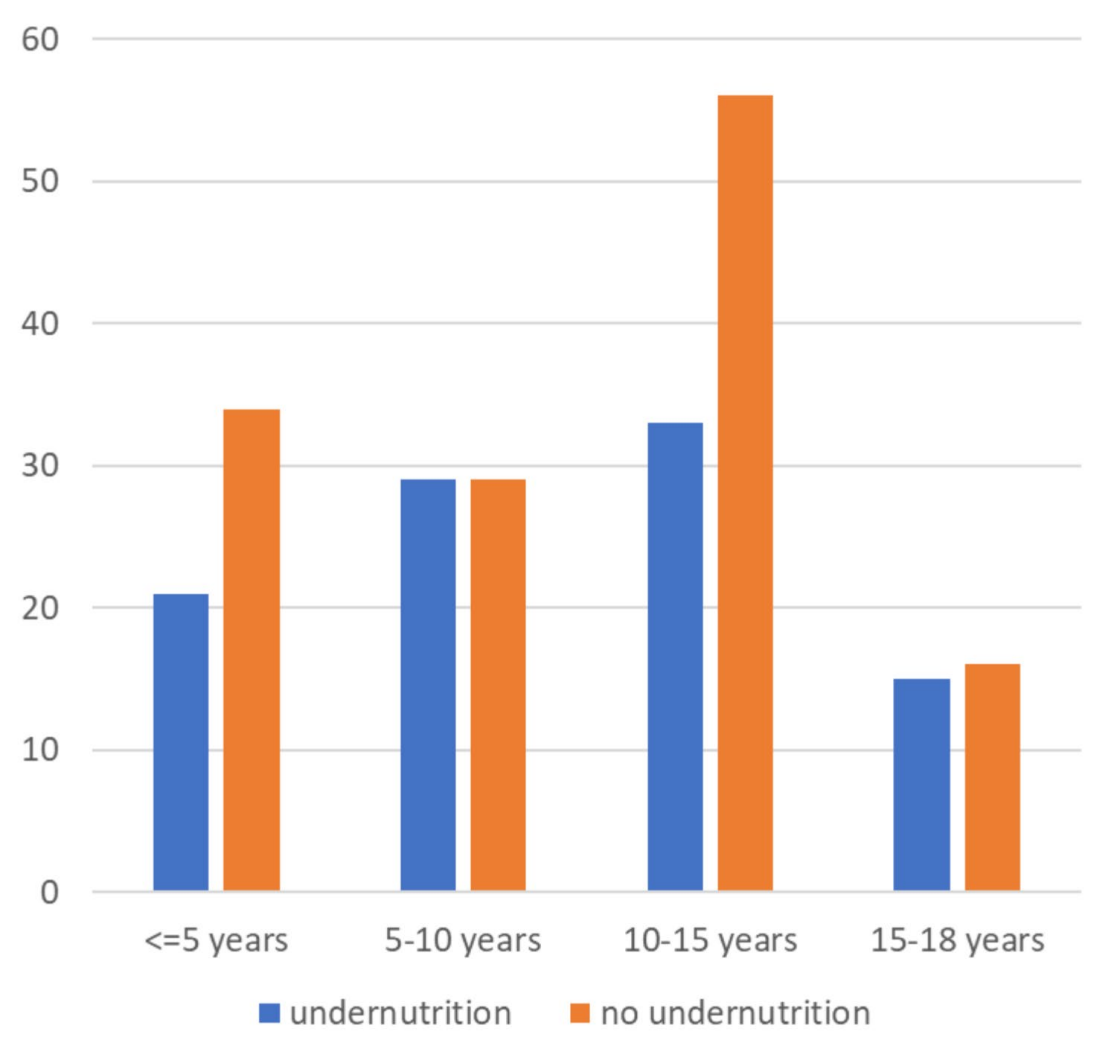
Overall undernutrition by age groups

**Fig. 2:**
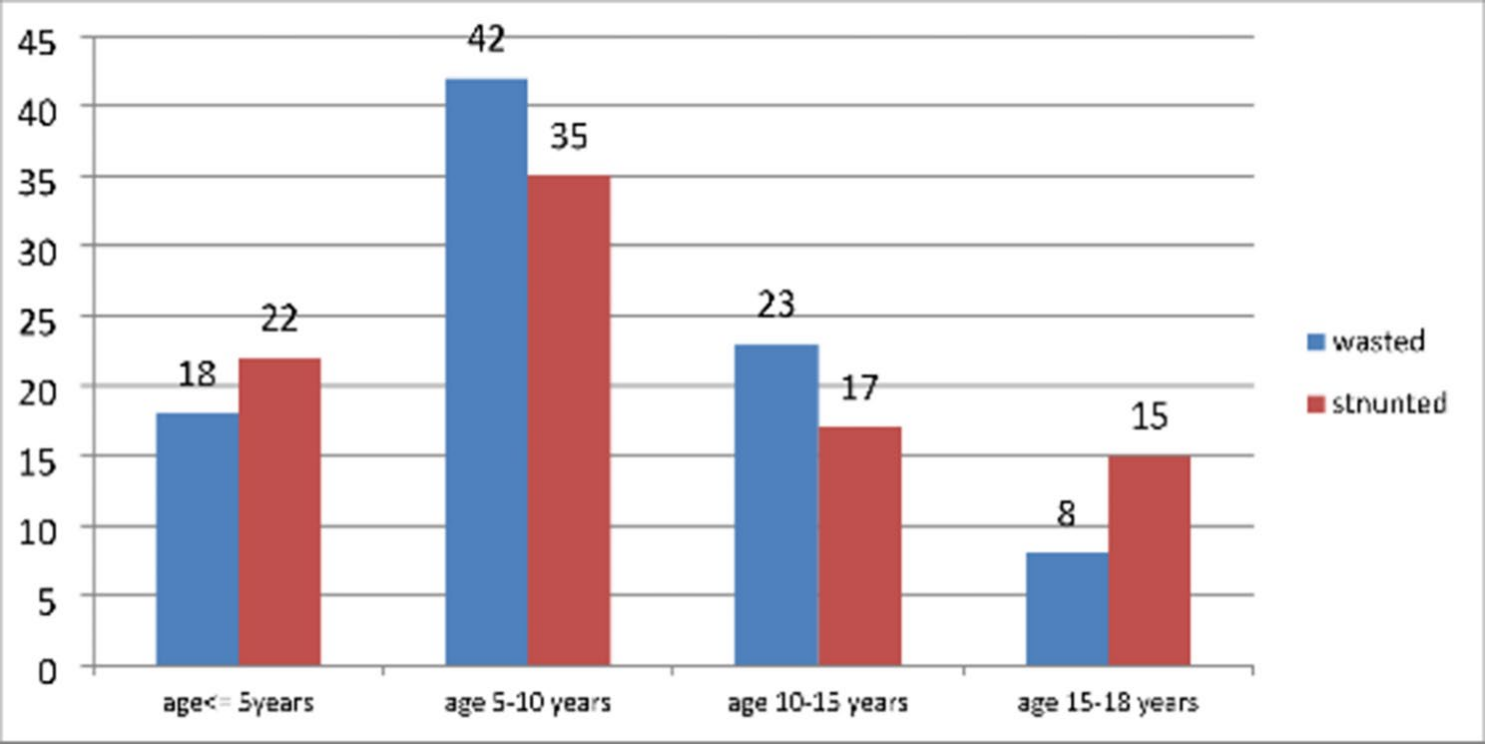
Proportions of wasting and stunting along with different age groups

The overall magnitude of undernutrition was 141(59%) of which 89(63.1%) had moderate to severe stunting, 91(64.5%) moderate to severe wasting, and 39(27.7%) had both. The proportion of undernutrition by age is depicted in **(**Figs. 1 and 2**).**

### Factors associated with undernutrition among children with epilepsy

From the Multivariable logistic regression sex of the child, paternal level of education, presence of delay in motor development, and gum hyperplasia were significantly associated with undernutrition. Males with epilepsy were 1.96 times at high risk for undernutrition as compared to females (AOR = 1.96; 95%CI: 1.04, 3.69). The odds of undernutrition among children with epilepsy whose fathers had no formal education were 1.88 times higher as compared to those whose fathers had at least primary school level education (AOR = 1.88,95%CI: 1.01,3.50). Children with epilepsy who had delay in motor development had 5.91 times higher odds of undernutrition as compared to those who had normal motor development (AOR = 5.91; 95%CI: 1.55,22.49). Whereas children with gum hyperplasia had 68%lesser risk of undernutrition than those who had no gum hyperplasia (AOR = 0.32; 95%CI: 0.12, 0.81). (Table 4)

**Table 4:** Binary and multivariable Logistic analysis of factors associated with malnutrition among epileptic patients at GUSCH pediatric neurology follow-up clinic, 2021(n = 239)

Variable	Category	Undernutrition		COR	AOR	p-value
		Yes	No			
Age	<=5 years	34	21	1.51 (0.62–3.70)	0.40(0.07–2.30)	0.31
	5–10 years	56	29	1.81 (0.79–4.17)	0.77(0.185–3.16)	0.71
	10-15years	35	33	0.99(0.43–2.33)	0.73(0.25–2.12)	0.57
	15-18years	15	15		1	.
Sex	Male	55	96	0.51(0.02–1.04)	1.96(1.05–3.69)	0.04
	Female	43	45		1	.
Paternal education	No formal education	69	36	1.65(0.97–2.79)	1.88(1.01–3.50)	0.05
	Primary and above	72	62		1	.
Motor developmental delay	No	102	88	3.36(1.59–7.13)	1	.
	Yes	39	10		5.91(1.55–22.49)	0.01
Language developmental delay	No	97	77	1.66(0.91–3.03)	1	.
	Yes	44	21		0.40(0.11–1.38)	0.15
Cognitive developmental delay	No	97	78	1.77(0.96–9.25)	1	.
	Yes	44	20		0.85(0.22–3.24)	0.81
Age at diagnosis	<=1 years	33	15	0.58(0.28–1.20)	1	.
	1–5 years	52	41	0.86(0.38–1.95)	0.55(0.21–1.43)	0.22
	5–10 years	38	20	0.37(0.16–0.89)	0.79(0.23–2.75)	0.71
	>=10 years	18	22		0.28(0.05–1.49)	0.14
Duration after diagnosis of epilepsy	<=1 years	33	15	0.58(0.28–1.20)	1	.
	1–5 years	52	41	0.86(0.38–1.95)	0.71(0.33–1.55)	0.39
	5–10 years	38	20	0.37(0.16–0.89)	0.58(0.17-2.00)	0.39
	>=10 years	18	22		0.29(0.04–2.02)	0.21
Comorbidity	No	105	90	3.86(1.71–8.72)	1	.
	Yes	36	8		2.79(0.88–8.82)	0.08
Recent admission	No	105	91	1.90(0.76–4.75)	1	.
	Yes	18	7		1.79(0.59–5.37)	0.30
Phenytoin	No	43	39	1.51(0.88–2.59)	1	.
	Yes	98	59		1.42(0.67-3.00)	0.36
Valproic acid	No	119	74	0.57(0.30–1.09)	1	.
	Yes	22	24		0.58(0.23–1.43)	0.23
Gum hypertrophy	No	135	89	0.44(0.15–1.28)	1	.
	Yes	13	20		0.32(0.12–0.81)	0.02
Depression	No	128	78	0.40(0.19–0.84)	1	.
	Yes	6	9		0.55(0.15–2.06)	0.38

List of Figures

## Discussion

This is the first report from a cross-sectional study in Ethiopia that showed the high magnitude of undernutrition and associated factors such as being male, low paternal educational status, presence of delay in motor development, and gum hyperplasia in epileptic children. This finding will help healthcare providers deliver risk-tailored nutritional advice and intervention. It will also serve as a baseline for further studies.

The prevalence of undernutrition in children with epilepsy was 59%. Of which 64.5% were having moderate to severe wasting, 63.1% had moderate to severe stunting, and 27.7% had both.

The finding in our study is higher than the findings in a cross-sectional study done in Turkey (13.8%), and a case-control study done in Norway (10.1%). This difference can be attributed to the socio-economic and cultural differences between the study populations. Children with epilepsy in developing countries are usually outcast from society, and some food items are excluded from the diets of epileptic patients. Moreover, prevention, screening and treatment of nutritional disorders in children with epilepsy and overall epileptic care in affluent nations’ settings are much better than the care in Ethiopia.[7, 9, 10, 13–16].

The finding in our study is higher than the finding in a database consolidating the result of 13 epidemiologic studies of epileptic children done in sub-Saharan African countries (25.4%) which included a larger sample size that might cause a significant difference in the magnitude of undernutrition between the two studies.

However, our finding was lower than the finding in a population-based case-control study done in Bangladesh among children with epilepsy aged less than 18 years where the prevalence of underweight and stunting was 75.9%and 79.4% respectively. This could be due to the difference in the anthropometric parameter used in the study. We employed BMI/age and/or MUAC/ age while the study in Bangladesh used weight/age to define wasting. There was also a study population difference, we included adolescents up to the age of 18 while they studied only children under the age of 10 years [15].

**Fig. 3:**
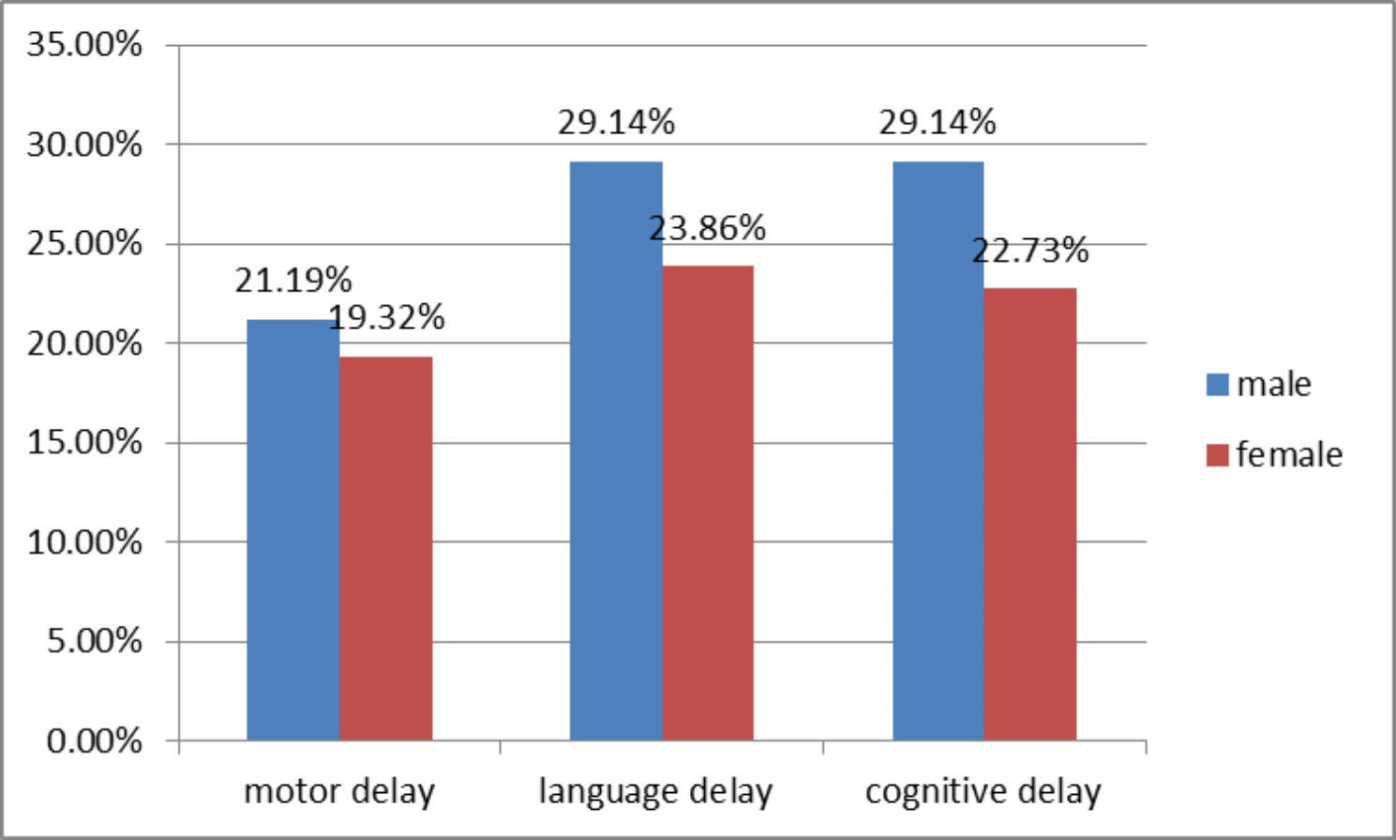
Distribution of developmental delay across sex

Male children with epilepsy were nearly two times at high risk for undernutrition as compared to females. This could be due to the higher prevalence of developmental delay among males in this study (Fig. 3). Most Ethiopian male children usually stay outdoors which can affect the frequency of feeding and increase the risk of undernutrition as compared to females who usually stay indoors. But a case-control study of Anthropometric Profile and Nutritional Status in Children with Generalized Epilepsy done in India showed no difference in nutritional status between genders [9].

The odds of undernutrition among epileptic children whose fathers had no formal education were about twice higher as compared to those whose fathers had at least primary school level education. This is supported by a population-based case-control study done in Bangladesh (AOR = 1.5 95%CI:1.0,2.1)[15]. This could be because caregiver knowledge and awareness of nutritional practices affect the nutritional status of a child[17]. Fathers are usually the backbones and breadwinners of the family in Ethiopia. Hence, the level of educational status of fathers might have an impact on the socioeconomic status of the family.

Epileptic Children with delay in motor development had six times higher odds of undernutrition as compared to those who have normal motor development. which is supported by nutritional assessments done in Italy and Bangladesh [15, 18]. This can be explained by feeding (chewing and swallowing) difficulties among children with motor disabilities which invariably affects the oropharyngeal neuromuscular structures.

Children with gum hyperplasia had a lesser odd of undernutrition as compared to those who had no gum hyperplasia. We have no evidence to support this finding. On the contrary, evidences showed that gum hyperplasia is positively associated with undernutrition[7].

## Conclusion

The study result showed that the magnitude of malnutrition was significantly high among epileptic children at GUCSH pediatric neurology follow-up clinic. Being Female sex and having gum hypertrophy was associated with malnutrition while low father educational level and presence of motor developmental delay were found positively associated with increased prevalence of malnutrition.

## Recommendation

Nutritional screening and intervention are recommended to be part of routine epileptic care, especially for those children with epilepsy with concomitant motor developmental delay. Further studies are recommended to determine the association between gum hyperplasia and nutritional status.

## Limitation of the study

As this study was done with a small number of patients visiting the pediatric neurology clinic, it may not tell the true figure of undernutrition in the general population. It also didn’t include the nutritional balance of the patient. The fact that it is a cross-sectional study may not show us the real effect of epilepsy on nutritional status.

## Data Availability

Data is available from the corresponding author upon reasonable request.

## Abbreviations

AOR: Adjusted Odds Ratio.
BMI: Body Mass Index.
CI: Confidence Intervals.
COR: Crude Odds Ratio.
ENA for SMART: Emergency Nutritional Assessment for Standardized Monitoring and Assessment of Relief and Transition.
HFA/LFA: Height for Age /Length for Age.
MUAC: Mid Upper Arm Circumference;
UOGCSH: University of Gondar Comprehensive and Specialized Hospital.
WHO: World Health Organization.
